# Deciphering the role of lipid metabolism-related genes in Alzheimer’s disease: a machine learning approach integrating Traditional Chinese Medicine

**DOI:** 10.3389/fendo.2024.1448119

**Published:** 2024-10-23

**Authors:** KeShangJing Wu, QingSong Liu, KeYu Long, XueQing Duan, XianYu Chen, Jing Zhang, Li Li, Bin Li

**Affiliations:** Department of Geriatrics, Hospital of Chengdu University of Traditional Chinese Medicine, Chengdu, Sichuan, China

**Keywords:** bioinformatics, Alzheimer’s disease, lipid metabolism, immune microenvironment, machine learning, Traditional Chinese Medicine

## Abstract

**Background:**

Alzheimer’s disease (AD) represents a progressive neurodegenerative disorder characterized by the accumulation of misfolded amyloid beta protein, leading to the formation of amyloid plaques and the aggregation of tau protein into neurofibrillary tangles within the cerebral cortex. The role of carbohydrates, particularly apolipoprotein E (ApoE), is pivotal in AD pathogenesis due to its involvement in lipid and cholesterol metabolism, and its status as a genetic predisposition factor for the disease. Despite its significance, the mechanistic contributions of Lipid Metabolism-related Genes (LMGs) to AD remain inadequately elucidated. This research endeavor seeks to bridge this gap by pinpointing biomarkers indicative of early-stage AD, with an emphasis on those linked to immune cell infiltration. To this end, advanced machine-learning algorithms and data derived from the Gene Expression Omnibus (GEO) database have been employed to facilitate the identification of these biomarkers.

**Methods:**

Differentially expressed genes (DEGs) were identified by comparing gene expression profiles between healthy individuals and Alzheimer’s disease (AD) patients, using data from two Gene Expression Omnibus (GEO) datasets: GSE5281 and GSE138260. Functional enrichment analysis was conducted to elucidate the biological relevance of the DEGs. To ensure the reliability of the results, samples were randomly divided into training and validation sets. The analysis focused on lipid metabolism-related DEGs (LMDEGs) to explore potential biomarkers for AD. Machine learning algorithms, including Support Vector Machine-Recursive Feature Elimination (SVM-RFE) and the Least Absolute Shrinkage and Selection Operator (LASSO) regression model, were applied to identify a key gene biomarker. Additionally, immune cell infiltration and its relationship with the gene biomarker were assessed using the CIBERSORT algorithm. The Integrated Traditional Chinese Medicine (ITCM) database was also referenced to identify Chinese medicines related to lipid metabolism and their possible connection to AD. This comprehensive strategy aims to integrate modern computational methods with traditional medicine to deepen our understanding of AD and its underlying mechanisms.

**Results:**

The study identified 137 genes from a pool of 751 lipid metabolism-related genes (LMGs) significantly associated with autophagy and immune response mechanisms. Through the application of LASSO and SVM-RFE machine-learning techniques, four genes—choline acetyl transferase (CHAT), member RAS oncogene family (RAB4A), acyl-CoA binding domain-containing protein 6 (ACBD6), and alpha-galactosidase A (GLA)—emerged as potential biomarkers for Alzheimer’s disease (AD). These genes demonstrated strong therapeutic potential due to their involvement in critical biological pathways. Notably, nine Chinese medicine compounds were identified to target these marker genes, offering a novel treatment approach for AD. Further, ceRNA network analysis revealed complex regulatory interactions involving these genes, underscoring their importance in AD pathology. CIBERSORT analysis highlighted a potential link between changes in the immune microenvironment and CHAT expression levels in AD patients, providing new insights into the immunological dimensions of the disease.

**Conclusion:**

The discovery of these gene markers offers substantial promise for the diagnosis and understanding of Alzheimer’s disease (AD). However, further investigation is necessary to validate their clinical utility. This study illuminates the role of Lipid Metabolism-related Genes (LMGs) in AD pathogenesis, offering potential targets for therapeutic intervention. It enhances our grasp of AD’s complex mechanisms and paves the way for future research aimed at refining diagnostic and treatment strategies.

## Introduction

Alzheimer’s disease (AD), termed the most prevalent type of dementia, affects one new person every three seconds globally. By 2026, there will be 13.8 million people in the US with Alzheimer’s disease (1). Despite various hypotheses, the underlying mechanisms of AD remain elusive. AD imposes a tremendous social, psychological, and economic impact on society, but no effective treatment is available to stop or reverse its course. Over the course of several decades of research, intricate connections have been unveiled between lipid metabolism and crucial pathological mechanisms of AD. These mechanisms include amyloidogenesis, bioenergetic insufficiency, oxidative stress, neuroinflammation, and myelin degradation (2). These multifaceted interactions provide a mechanistic understanding of the intricate pathogenesis of AD and represent promising avenues for therapeutic intervention. However, how lipid metabolism genes play an identifying role in AD has not yet been elucidated, and this study aims to fill that gap Some of these biomarkers may have prognostic or diagnostic significance.

Lipids are essential in maintaining the structural and functional aspects of the body, including their significance in neurodegenerative diseases such as AD. In the brain, various lipid classes, including sphingolipids, glycerophospholipids (GPs), and cholesterol, are present in high concentrations (3). Cholesterol, a major component of cellular membranes, is crucial in sustaining the structural integrity of neuronal membranes (4). GPs and sphingolipids perform dual roles by contributing to neuronal membrane integrity and serving as precursors for synthesizing bioactive lipid mediators in the brain. Considering the aforementioned functions, lipid metabolism may be closely associated with the progression and treatment of AD.

The molecular mechanisms underlying AD remain elusive, but a hallmark feature of the disease is the increase in β-amyloid (Aβ) and tau protein aggregates in the brain (5). AD is primarily associated with mutations in three genes, with the amyloid precursor protein gene (APP) playing a vital role (6). A key genetic determinant of AD susceptibility is apolipoprotein E (APOE), which has three common isoforms with distinct impacts on lipid metabolism and Aβ dynamics. Besides APOE, several other genes implicated in lipid metabolism, such as ATP-binding cassette subfamily A member 1 (ABCA1), ATP-binding cassette subfamily A member 7 (ABCA7), and phospholipase D3 (PLD3), have been associated with AD risk (7).The above shows the complexity of the pathological development process of AD. Studies have now concluded that The APOE ϵ4 genotype, a major genetic risk factor for AD, is involved in lipid transport and metabolism, influencing the disease’s progression (8). Lipidomic profiles can distinguish between individuals with and without cognitive impairment, suggesting potential for early diagnostic biomarkers (9) Although there are some important intersections between lipid metabolism genes and the pathological process of AD, the specific process explaining how lipid metabolism-related genes (LMGs) affect the production, degradation, transport, and aggregation of Aβ in the brain and thus regulate AD pathogenesis has not been fully uncovered.

Traditional Chinese herbs can regulate lipid absorption, reduce lipid synthesis, and enhance lipolysis and lipid transport. It is important to note that Chinese medical theory has a long history of using herbal medicine to treat diseases associated with lipid metabolism, such as Phlegm disease. A study revealed the potential of certain herbal treatments, such as Areca catechu, Acorus calamus, and Eclipta alba, among others, in enhancing memory and cognitive functions in patients with AD (10). BSTSF may protect against Alzheimer’s disease by restoring metabolic balance in the brain, potentially benefiting cognitive performance and lipid metabolism (11). Therefore, exploring chinese medicines that modulate the targets related to lipid metabolism for the treatment of AD provides new preventive and therapeutic strategies. Several studies have shown that the occurrence of AD is related to lipid metabolism. However, although studies have now revealed the relationship between some LMGs and Alzheimer’s pathology or diagnosis, more links between LMGs and their associations need to be revealed. Alzheimer’s disease (AD) is a multifactorial neurodegenerative disorder characterized by complex pathological mechanisms, including amyloid-beta plaque formation, tau protein tangles, neuroinflammation, and lipid metabolism dysregulation. Despite decades of research, the intricate nature of AD has made it difficult to identify reliable biomarkers for early diagnosis and effective therapeutic targets. Traditional approaches often fall short in capturing the full spectrum of molecular interactions underlying disease progression. This complexity underscores the need for more advanced techniques, such as machine learning. This research focused on extracting gene expression data related to lipid metabolism in the context of AD. The process involved utilizing the LASSO and SVM-RFE approaches to identify AD diagnostic markers related to lipid metabolism (12). Furthermore, the related mechanisms of action were assessed by Gene-Set Enrichment Analysis (GSEA) and Gene-Set Variation Analysis (GSVA).

**Figure 1:**
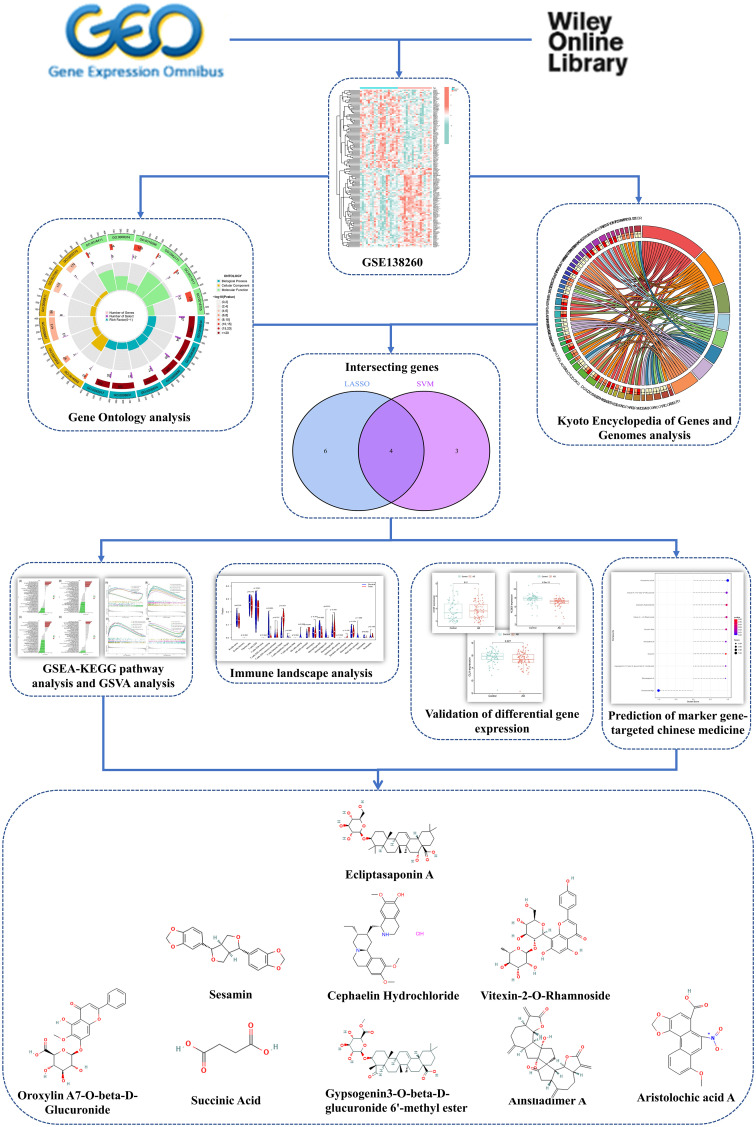
Illustration of the research approach.

Additionally, the study identified Chinese Medicine drugs that specifically target these biomarkers and provided insights into the regulatory correlation involving these genes in the ceRNA network. This study aimed to investigate the potential gene biomarkers along the AD based on LMGs. **Figure 1** illustrates the research approach employed in this study.

## Materials and methods

### Data source

The GEO database was searched to obtain the expression data of the AD-related genes and normal samples. The GSE138260 dataset, comprising a total of 36 samples (17 AD and 19 normal), was used as the training set. Furthermore, encompassing 74 normal and 87 AD samples, the GSE5281 was utilized to verify the marker gene expression. Moreover, the LMGs (n = 751) utilized were acquired from a referenced source and functioned as the foundation for subsequent analyses in this research (13). The detailed genes are summarized in **Supplementary Table 1**.

### Differential expression analysis

Extracting the expression of lipid metabolism genes from the GSE138260 dataset and performing differential analysis of lipid metabolism genes by limma package were the steps involved in this process. To identify the differentially expressed genes (DEGs) between these two sample groups, R software was utilized. Using a P-value cutoff lower than 0.05, DEGs were filtered between the AD and control groups (**Supplementary Table 2**). These thresholds were deemed appropriate for screening and selecting genes that were significantly differentially expressed in AD.

### Functional enrichment performed in Metascape

Functional analyses of the DEGs from this study were executed via Metascape, a web-based tool (http://metascape.org/). Analyses such as Gene Ontology (GO), Reactome pathway enrichment, and Immunologic Signatures enrichment were conducted to elucidate the possible functions that these DEGs may be involved in. Notably, the immunologic signatures enrichment analysis utilized the database Immunologic Signatures in this research. This database is constructed by integrating immune-related enrichment analysis of the target gene from published literature. Reactome, on the other hand, is a comprehensive database of expert-curated and peer-reviewed articles covering a wide range of reactions and biological pathways in humans.

### Identification of optimal diagnostic gene biomarkers for AD

The glmnet package was employed to execute feature selection using the LASSO algorithm (14, 15). DEGs between individuals with AD and healthy individuals were utilized to identify gene biomarkers for AD. Additionally, a SVM-RFE model was developed with the package SVM, with a comparative assessment of the average misjudgment rates of their 10-fold cross-validations (16). The markers acquired from the above-mentioned two approaches were overlapped to determine the optimal AD gene markers. The receiver operating characteristic (ROC) curve and the area under the curve (AUC) were measured to determine the capacity of these screened markers in diagnosing the disease. Additionally, accuracy, sensitivity, and specificity were derived to assess the diagnostic ability. Furthermore, for predicting sample types in GSE138260, a four-marker gene-based logistic regression model was developed using the “predic” function of R glm. The diagnostic capacity of the model was evaluated by assessing the ROC curves.

### Single-gene GSEA

The underlying pathways associated with the four marker genes were examined in-depth through R GSEA (V.4.1.0). The association of all other genes in GSE138260 with the marker genes was assessed. The resulting gene set was then ranked according to their correlations from highest to lowest and subsequently tested for enrichment with the signaling pathway KEGG defined as a predefined set. This was followed by integrating the enrichment results specific to every marker gene into the analysis (**Supplementary Table 3**).

### Single-gene GSVA

This research utilized the R GSVA (V.1.38.0) to analyze the gene set variation (17). The background gene set was considered the KEGG set, with the analysis executed on every marker gene. The package limma comparatively assessed the GSVA score of the samples in the expression groups (high and low) for each marker gene. The screening criteria for identifying significant differences were set as |t| >2 and *P* < 0.05. For cases where t > 0, the pathway was considered activated in the high-expression group. Conversely, when t < 0, the pathway was deemed activated in the low-expression group. This was followed by integrating the enrichment results specific to every marker gene (**Supplementary Table 4**).

### Prediction of potential Chinese medicines and ingredients with therapeutic effects

The Integrated Traditional Chinese Medicine (ITCM) (http://itcm.biotcm.net) was used to find key gene-related Chinese medicines and linked components. Initially, the components of Chinese medicines were matched with the related components from the key genes. Hence, it allowed the screening and determination of potential therapeutic options by identifying Chinese medicines that share similar components with the key genes through the matching process.

### Immune infiltration analysis

CIBERSORT is a powerful tool used to decipher the cellular composition of complex tissues per gene expression profiles (18). CIBERSORT was employed to predict the abundance of 22 different types of infiltrating immune cells within the tissue samples obtained from GSE138260 (**Supplementary Table 5**). The proportions of each immune cell type were calculated for every sample, and the sum of all the fractions of the assessed immune cell types was equal to 1 (19). This analysis facilitated the elucidation of the immune cell landscape in AD and the identification of potential immune cell targets for further investigation.

### Construction of ceRNA network

Using starBase, the mRNA-miRNA interaction pairings were predicted based on the four marker genes. The National Center for Biotechnology Information (NCBI) and miRbase were used to retrieve the corresponding RNA sequences and miRNA data. The threshold score for binding was adjusted to 170 (the default was 140) when the mRNA-miRNA nucleic acid binding was predicted using the Miranda program. The projected miRNA was then evaluated using starBase, and the relationships between miRNA and lncRNA were filtered to create the ceRNA network of mRNA-miRNA-lncRNA.

### Statistical analysis

The statistical analyses in this study involved several methods. The student’s t-test comparatively assessed the variation across the groups, while Pearson correlation analysis was employed to assess the relationship among the 137 DEGs. The Venn Diagram package was employed to create the Venn diagram for visualizing the ceRNA network. Additionally, Cytoscape was utilized to generate visualizations of the network. *P* < 0.05 indicated the statistical significance level. The software R was utilized to execute all analyses.

## Results

### Identification of lipid metabolism-related DEGs

**Figure 2:**
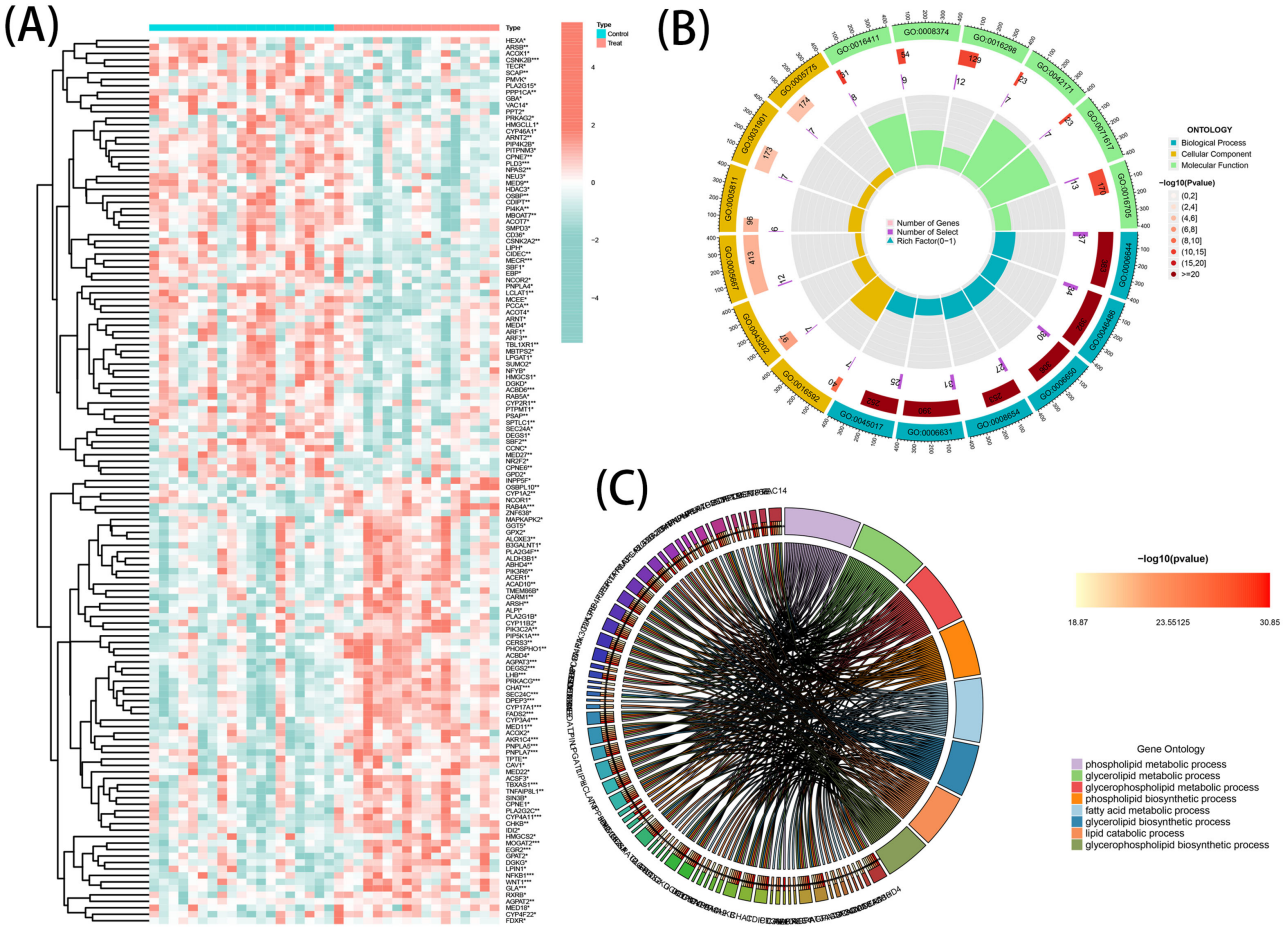
DE-LMGs expression levels, Gene Ontology (GO) and Kyoto Encyclopedia of Genes and Genomes (KEGG) in AD. **(A)** Violin plots show expression patterns of LMGs across samples. **(B)** Bubble diagram of GO enrichment function analysis. **(C)** Relationship among the top 8 enriched KEGG pathway terms and targets is represented in a chord plot.

A total of 137 DEGs were identified from GSE138260, consisting of 69 upregulated genes and 68 downregulated genes. The patterns exhibited by the DEGs expression among samples are presented in a clustering heatmap (**Figure 2A**). Notably, choline acetyl transferase (CHAT) was negatively associated with member RAS oncogene family (RAB4A) and acyl-coA binding domain-containing protein 6 (ACBD6). Meanwhile, a positive association of RAB4A with CHAT and ACBD6 was observed.

### Functional analyses of the DEGs

GO Enrichment and Reactome Pathway analyses were executed to comprehensively understand the biological roles and pathways of the DEGs. The resulting data of the analyses (GO and KEGG) indicated a strong association of the DEGs with multiple functional categories, including ‘phospholipid metabolic process’, ‘glycerolipid metabolic process’, and ‘fatty acid metabolic process’ (**Figure 2B**). Additionally, a circos graph was employed to depict the association of the KEGG results of the eight top terms with the associated differential genes (**Figure 2C**). These findings indicated the involvement of DEGs in the pathogenesis of AD and suggested that they may exert this function by regulating the metabolic processes of phospholipids, glycerolipids, and fatty acids.

### Four DEGs identified as AD diagnostic genes

**Figure 3:**
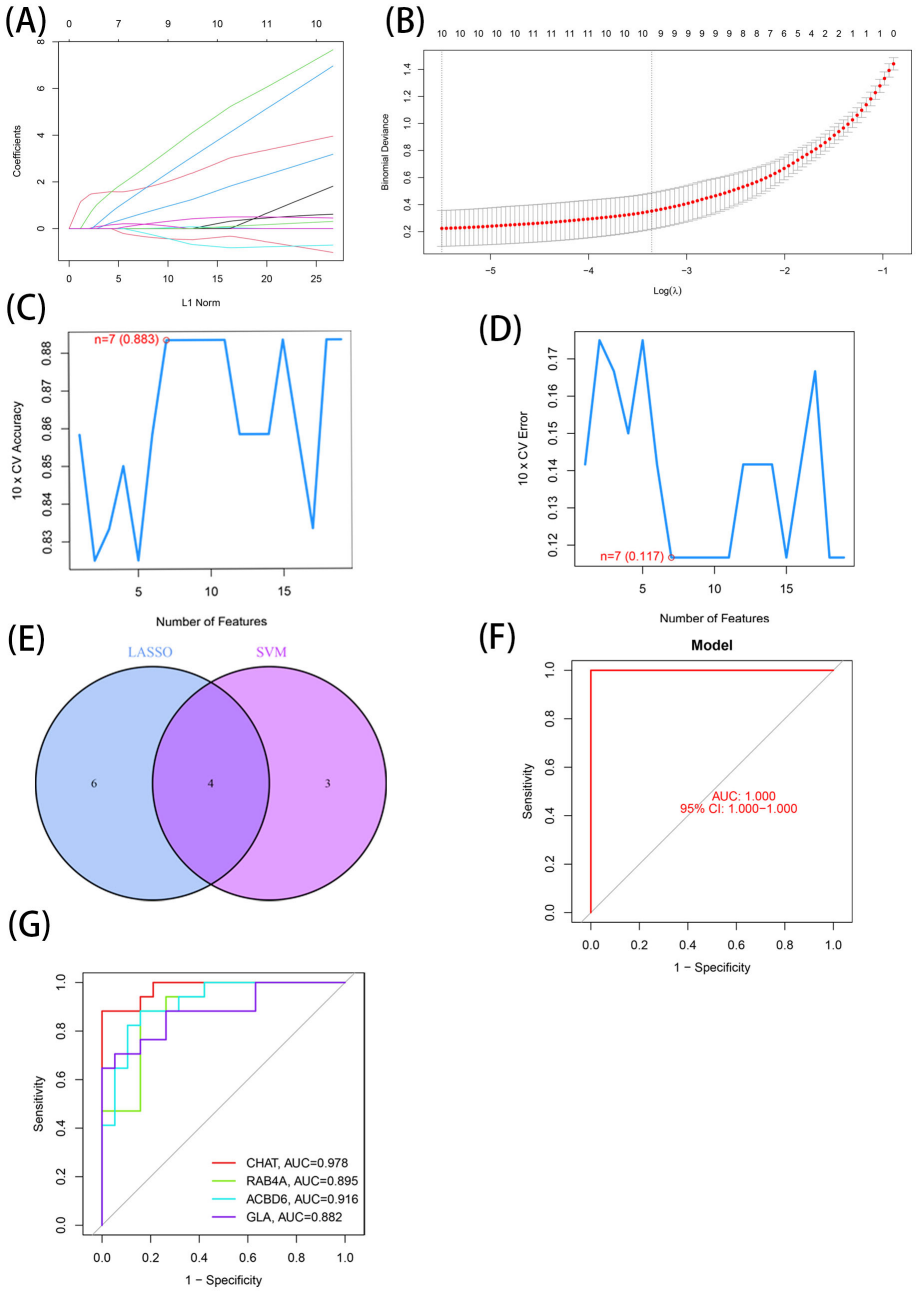
7 LMDEGs were identified as diagnostic genes for AD. **(A, B)** By LASSO logistic regression algorithm, with penalty parameter tuning conducted by 10-fold cross-validation, was used to select 12 AD-related features. **(C, D)** SVM-RFE algorithm to filter the 137 DE-LMGs to identify the optimal combination of feature genes. Finally, 4 genes ((maximal accuracy =0.883, minimal RMSE =0.117)) were identified as the optimal feature genes. **(E)** The marker genes obtained from the LASSO and SVM-RFE models. **(F)** Logistic regression model to identify the AUC of disease samples. **(G)** ROC curves for the 4 marker genes.

The DEGs identified through the comparative assessment of individuals with AD and healthy individuals were assessed concerning their diagnosis capacity. The process involved utilizing LASSO and SVM-RFE in the GSE20680 dataset. Four characteristics connected to AD were identified using the logistic regression technique, LASSO with penalty parameter adjustment carried out by 10-fold cross-validation (**Figures 3A, B**). Overall 12 such features (**Figures 3C, D**) were screened through this approach. SVM-RFE filtered the DEGs to identify the optimal combination of feature genes, yielding four marker genes (CHAT, RAB4A, ACBD6, and alpha-galactosidase A [GLA]) for subsequent analysis (**Figure 3E**).

Based on these four marker genes (maximal accuracy =0.883, minimal RMSE =0.117), a logistic regression model was established, which exhibited excellent performance in distinguishing AD from normal samples with an AUC of 1 (**Figure 3F**). ROC curves were developed for the four marker genes, demonstrating that the individual genes were also highly accurate in differentiating AD samples from normal samples, with an AUC greater than 0.8 for all genes (**Figure 3G**). These results suggest that this model outperformed the individual marker genes in terms of accuracy and specificity when distinguishing between AD and normal samples.

### Linkage of marker genes to various AD-linked pathways

**Figure 4:**
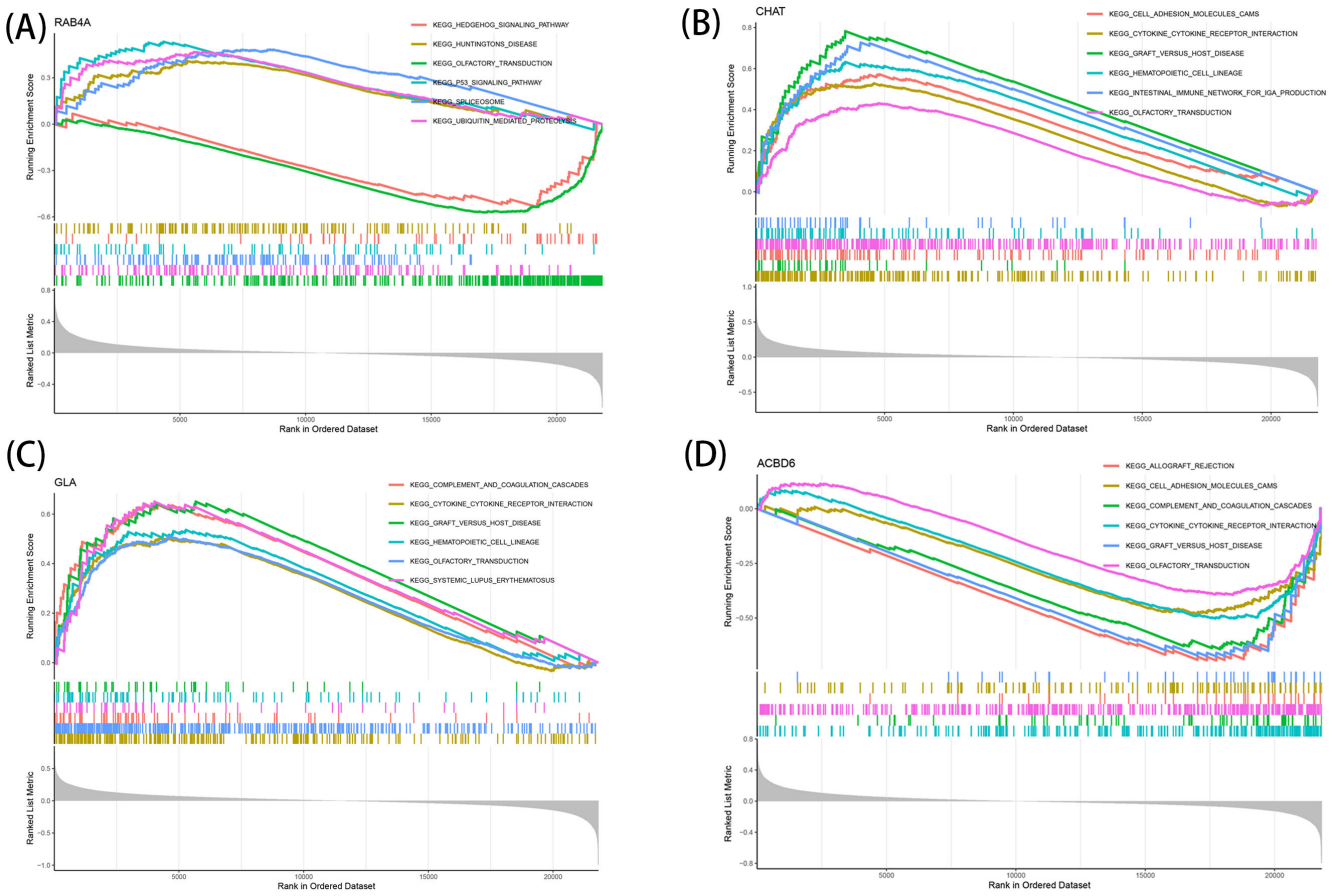
Single-gene GSEA-KEGG pathway analysis in RAB4A **(A)**, CHAT **(B)**, GLA **(C)**, ACBD6 **(D)**.

**Figure 5:**
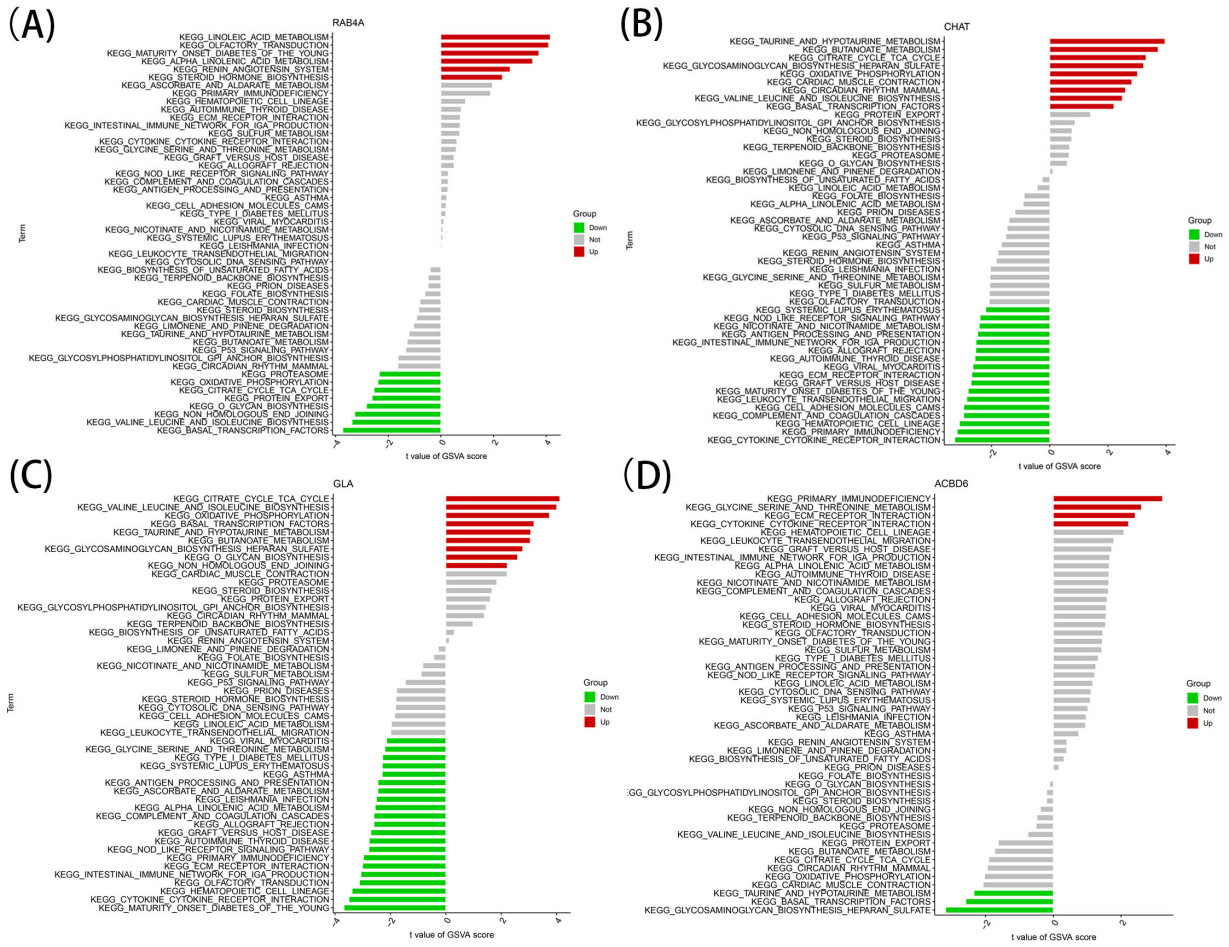
High and low-expression groups based on the expression levels of each marker gene combined with GSVA in RAB4A **(A)**, CHAT **(B)**, GLA **(C)**, ACBD6 **(D)**.

The identified marker genes were assessed concerning their possible involvement in AD through a comprehensive single-gene GSEA-KEGG pathway analysis. **Figures 4A–D** exhibits the first six enriched pathways for every marker gene. The resulting data depicted the enrichment of these genes in various biological pathways, including Spliceosome, Hematological system (‘Complement and coagulation cascades’ and ‘Hematopoietic cell lineage’) and immune response (‘Hedgehog signaling pathway’, ‘Cell adhesion molecules [CAMs]’ and ‘Intestinal immune network for immune globulin antibody production’). Treatment methods (‘Ubiquitin mediated proteolysis’), and various disease pathways (‘Huntington’s disease’, ‘Systemic lupus erythematosus’, and ‘Graft versus host disease’) also depicted such enrichment. Moreover, the resulting data showed that these marker genes were enriched in the ‘Cytokine receptor interaction’. Furthermore, CHAT was closely related to the ‘Olfactory transduction’ (also enriched in ACBD6, RABA4, and GLA). In addition, the differentially active pathways in the two groups (high and low) were evaluated based on the levels of marker gene expression in association with GSVA. The results suggested that AD may be promoted by basal transcription factors by reducing ACBD6 expression in the illness, glycosaminoglycan biosynthesis, heparan sulfate, and taurine and hypotaurine metabolism. However, the overexpression of ACBD6 activated ‘PRIMARY IMMUNODEFICIENCY’, ‘GLYCINE SERINE AND THREONINE METABOLISM’, ‘ECM RECEPTOR INTERACTION’, and ‘CYTOKINE RECEPTOR INTERACTION’ (**Figure 5D**). The upregulation of RAB4A promoted the amino acid pathways (‘GLYCINE SERINE AND THREONINE METABOLISM’) (**Figure 5A**). Moreover, GLA, with inhibited expression in AD tissue, was more closely related to various diseases (‘SYSTEMIC LUPUS ERYTHEMATOSUS’, ‘AUTOIMMUNE THYROID DISEASE’ and ‘GRAFT VERSUS HOST DISEASE’), ‘REGULATION OF AUTOPHAGY’ and amino acid metabolism pathway (**Figure 5C**). However, the highly expressed CHAT stimulated the pathways, for instance, ‘TAURINE AND HYPOTAURINE METABOLISM’, ‘BUTANOATE METABOLISM’, ‘DILATED CARDIOMYOPATHY’, ‘CITRATE CYCLE TCA CYCLE’ and ‘CARDIAC MUSCLE CONTRACTION’ that may induce AD (**Figure 5B**).

### Immune landscape analysis

**Figure 6:**
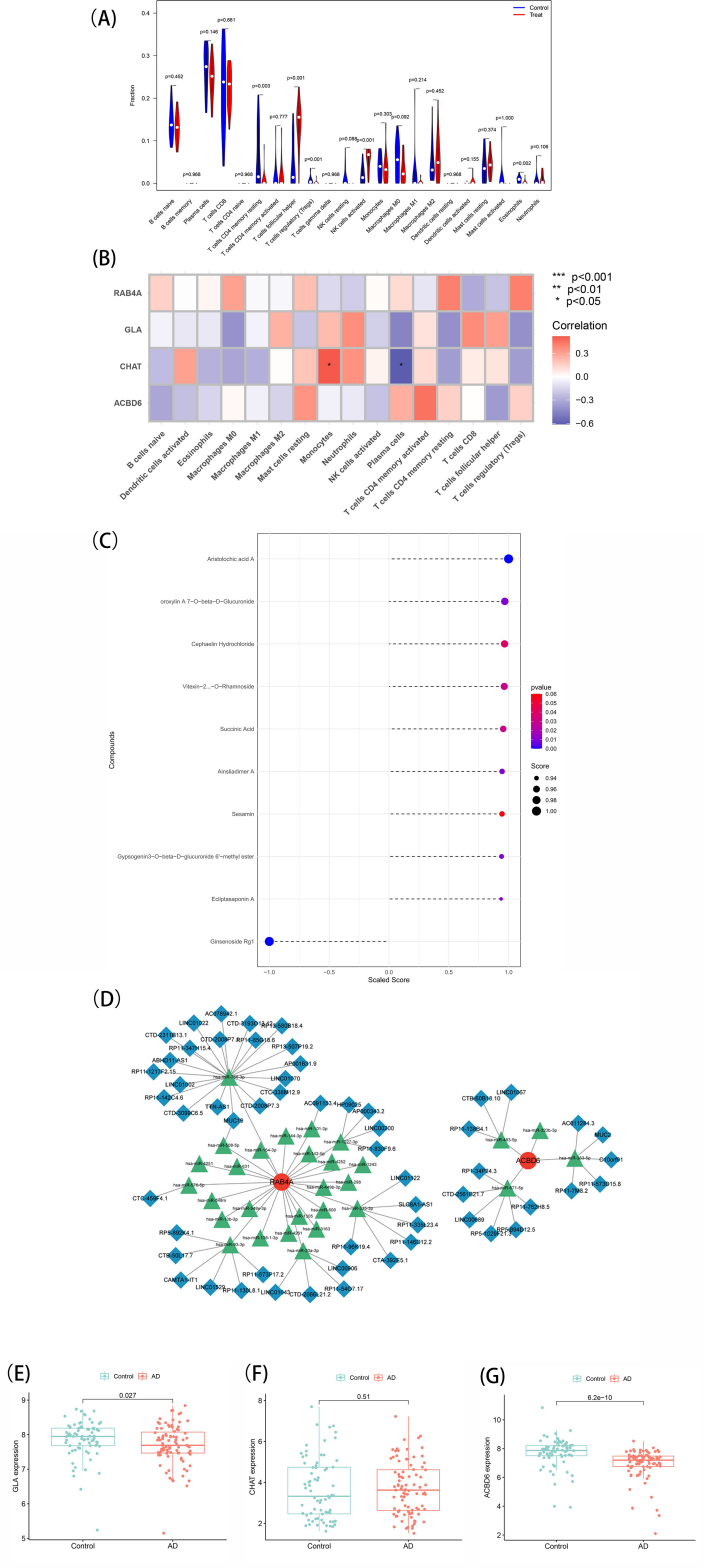
**(A)** Immune landscape analysis. Implemented the CIBERSORT algorithm to explore the differences in the immune microenvironment between AD patients and normal samples. **(B)** Pearson correlation analysis revealed that monocytes and plasma cells had strong positive and negative correlations with CHAT(*p < 0.05, **p < 0.01). **(C)** Prediction of marker gene-targeted chinese medicine. The chinese medicine may target marker genes through the ITCMDb database and the interaction relationship. A total of 9 potential chinese medicine were extracted. **(D)** A ceRNA networks based on marker genes. The network included 88nodes (2 marker genes, 29 miRNAs and 57 lncRNAs) and 86 edges. **(E, F, G)** Expression of the marker gene in the validation set. The expression of marker genes in the GSE5281 dataset.

In order to examine the association of the marker genes with the immune microenvironment in AD, the CIBERSORT algorithm was employed to explore the variance in immune cell populations between individuals with AD and normal samples. The results showed that the B-cell naïve fraction was lower in AD samples in comparison with normal samples (**Figure 6A**). In contrast, monocytes and T-cell follicular helper were more abundant in AD samples. Importantly, Pearson correlation analysis exhibited a strong positive association of CHAT with monocytes and a negative association with plasma cells. The resulting data imply that CHAT could be associated with the alterations in the immune microenvironment of individuals with AD (**Figure 6B**).

### Prediction of marker gene-targeted Chinese Medicine drugs

To identify potential therapeutic options for AD, the Integrated Traditional Chinese Medicine (ITCM) database was employed to identify drugs that targeted the marker genes. The results are shown in **Figure 6C**. A total of 9 Chinese Medicine were targeted. Among them, Aristolochic acid A, Oroxylin A7-O-beta-D-Glucuronide, Cephaelin Hydrochloride, Vitexin-2-O-Rhamnoside, Succinic Acid, Ainsliadimer A, Sesamin, Gypsogenin3-O-beta-D-glucuronide 6’-methyl ester, and Ecliptasaponin A were considered relevant. These findings suggest that drugs targeting the marker genes could be a promising therapeutic strategy for AD using Chinese Medicine.

### Marker genes-based ceRNA network

To explore the potential ceRNA network related to AD, starBase and miranda were assessed to develop the ceRNA network based on four marker genes. This network comprised 88 nodes, including two marker genes, 29 miRNAs, and 57 lncRNAs, connected by 86 edges (**Figure 6D**). The analysis indicated competitive binding of the 43 lncRNAs to various miRNAs and their involvement in regulating RAB4A. Of these, hsa-miR-766-3p and hsa-miR-508-5p were determined to be shared by 21 lncRNAs. Moreover, 22 shared lncRNAs could target hsa-miR-576-5p, hsa-miR-93-3p, hsa-miR-20a-3p, hsa-miR-335-3p, hsa-miR-1227-3p, and hsa-miR-101-3p, respectively. Concerning ACBD6, 13 lncRNAs were determined that could regulate its expression through binding competitively with hsa-miR-363-5p, hsa-miR-483-5p, hsa-miR-323b-3p, and hsa-miR-671-5p. Notably, hsa-miR-363-5p was found to bind to 4 lncRNAs to regulate ACBD6, with LINC01067 and LINC00689 targeting hsa-miR-483-5p and hsa-miR-671-5p simultaneously. ceRNA network characteristics are explored in **Supplementary Table 6**.

### Expression of the marker gene in the validation set

Finally, the levels of marker genes were assessed in GSE5281. The results showed that the CHAT, GLA, and ACBD6 expression patterns were congruent with the data acquired from the GSE138260 dataset. Specifically, CHAT (P = 0.51) was expressed more in AD patients with notably heightened levels than normal samples. In contrast, the expression of GLA (P = 0.027) and ACBD6 (P = 6.2e-10) was significantly lower in AD samples (**Figures 6E–G**). These findings provide further evidence that CHAT, GLA, and ACBD6 may hold promise as biomarkers for AD diagnosis and treatment.

## Discussion

AD, being a form of dementia, still lacks a clear understanding of its precise mechanisms, despite significant advancements in recent years. There has been some research into the disease, such as generational genetic studies. The research has implicated several pathways to be essential to the pathogenesis of AD, usually in cellular and animal models. Nonetheless, additional research is required to reach conclusive results. The diagnosis of the spectrum of AD at the clinical level involves three sets of criteria, including the National Institute on Aging-Alzheimer’s Association (NIA-AA) definition, the Diagnostic and Statistical Manual of Mental Disorders (DSM-5), and the International Working Group criteria, each of them including development disability and suspected dementia symptoms (20). The downside is that without significant cognitive impairment, neurological findings will generally appear normal in the initial stages.Consequently, distinguishing the disease from other conditions, including other causes of dementia, can be difficult (21). Indeed, the identification of a reliable diagnostic biomarker for AD is of great significance. Early and accurate diagnosis of AD facilitates timely interventions and treatments, maximizing the potential benefits for patients.

The normal individuals and individuals with AD based on GEO datasets were utilized to determine the 137 DEGs, with notable upregulation and downregulation exhibited by 69 and 68 genes, respectively. Enrichment was assessed through KEGG with strong associations exhibited with Spliceosome, Hematological system, and immune response. Amyloid beta (Aβ) is a key component of amyloid plaques, which are abnormal extracellular deposits commonly observed in the cerebral tissue of individuals with AD. Amyloid plaques are primarily composed of aggregated and misfolded forms of Aβ, which are proteolytic fragments derived from the larger amyloid precursor protein (APP), with Aβ40 and Aβ42 being its two most prevalent types consisting of 40 and 42 amino acids, respectively. These Aβ peptides are considered by-products of APP metabolism (22). The development of Aβ plaques in AD conforms to a characteristic pattern. Initially, these plaques tend to appear in specific regions of the brain, including the basal, temporal, and orbitofrontal neocortex. As the disease progresses, the plaques spread throughout other regions, such as the neocortex, hippocampus, amygdala, diencephalon, and basal ganglia. Amyloid pathogenesis is initiated with abnormal cleavage of APP (integral plasma membrane protein) through β-secretases (BACE1) and γ-secretases. This results in the formation of insoluble Aβ fibrils, which then oligomerize and diffuse into synaptic clefts, thereby interfering with synaptic signaling (23). Given the notable function of Aβ in AD, it is reasonable to hypothesize that the DEGs identified in this context would be enriched in neurological diseases, particularly AD.

The pathway enrichment analysis in the current research depicted that enriched pathways in AD samples include Spliceosome, Complement and coagulation cascades, Hematopoietic cell lineage, Hedgehog signaling pathway, CAMs, and so on. Among them, spliceosome influences cancer up to an extent. Research has shown that certain components of spliceosomes, including U1 snRNA, SF3B1, and U2AF1, may be related to tumors, which are critically involved in cancer progression (24, 25). A study indicates that chronic cerebral hypoperfusion (CCH) can simultaneously trigger the coagulation and complement cascade in a new Alzheimer’s disease mouse model brain, potentially expediting AD pathology. These findings shed light on how blood flow alterations impact the blood-brain barrier (BBB) dysfunction and amyloid beta clearance, thus providing valuable insights into the advancement of neurodegenerative disorders such as AD (26). The Sonic Hedgehog (SHH) signaling pathway is critically involved in various aspects of neurogenesis and neural patterning during the development of the central nervous system.

Moreover, it is suggested that SHH pathway dysregulation may contribute to the pathogenesis of aging-related neurodegenerative diseases, such as AD (27). CAMs may contribute to AD pathogenesis by affecting amyloid-β metabolism, neuroinflammation, and vascular changes, while also playing a key role in brain plasticity, learning, memory, and recovery after injury (28, 29). The pathway enrichment analysis of AD has strongly suggested that the hematological system and immune responses are integral components of the disease. These findings are consistent with clinical observations and have piqued considerable interest in exploring the role of immune cell infiltration in AD and identifying diagnostic biomarkers associated with the hematological system.

The filtration and identification of diagnostic biomarkers for AD involved machine learning algorithms. The identified DEGs were subjected to LASSO regression and SVM-RFE algorithms, resulting in the selection of only four overlapping features, CHAT, GLA, ACBD6, and RAB4A. Among them, the AUC values of CHAT and ACBD6 rank in the top 2. Cholinergic dysfunction, a key feature of the cerebral tissues of individuals with AD, is characterized by a severe decrease in the cholinergic enzyme choline acetyl transferase (CHAT), which is responsible for the biosynthesis of the cholinergic neurotransmitter acetylcholine (ACh) (30, 31). The GSAV analysis of CHAT further supported the activation of the hematopoietic cell lineage in the low-expression group.

Nonetheless, the exact function of CHAT in AD is yet to be understood. Research has proposed that CHAT could impact AD progression by regulating processes such as amyloid-beta production, neuroinflammation, synaptic plasticity, and neurogenesis (32, 33). Recent research has shown that CHAT activity and protein are present in extracellular fluids, including human plasma and cerebrospinal fluid (34). A study presented evidence that the genetic variability of the CHAT locus can affect the levels of CHAT activity in plasma in individuals with AD. It also proposed that rivastigmine treatment could be associated with higher variability in plasma concentrations of both CHAT protein and activity in comparison to untreated AD patients (35). Apart from this, a meta-analysis revealed that a particular variant of CHAT is associated with a lower risk of AD and proposed that future research should examine the role of these genetic variants in AD susceptibility, as well as their potential utility as biomarkers for early diagnosis and prevention of AD (36). Therefore, the level of CHAT in peripheral blood is useful for the early diagnosis of AD, as well as for the early treatment and prevention of AD by detecting CHAT levels.

The significance of ACBD6 is highlighted by the association of its genetic mutations with the development of neurodegenerative disorders, including cognitive impairment, in humans. These mutations hinder the production of a functional protein, emphasizing the vital role of ACBD6 in regular physiological processes (37). The Acyl-CoA binding domain-containing proteins (ACBDs) represent a diverse family of proteins consisting of multiple genes, characterized by the existence of an 80-residue acyl-CoA binding motif (ACB), which is conserved (38, 39). Moreover, it has been proposed that ACBD6 is involved in regulating cellular metabolism through various regulatory pathways, as indicated by the observation of its invertebrate homologs. Furthermore, anox expression is localized in both chemosensory organs and central nervous system neurons and is necessary for modulating sugar-induced nerve responses and regulating insulin signaling (40). Homozygous mutations in the ACBD6 gene have been associated with neurodevelopmental disorders such as mental retardation, autosomal recessive microcephaly, etc., leading to intellectual disability as the lack of myristoylation deficiency affects brain functions (37).

Moreover, ACBD6 is an indispensable participant in acyl-CoA-dependent modification pathways that regulates the lipid and protein composition of human cell membranes, specifically by engaging in myristoylation processes and regulating lysophospholipid acyltransferase enzymes (LPLAT) (41). Data reports that ACBD6 exhibits adaptive binding properties that prevent its continuous saturation by highly abundant C 16:0 -CoA and safeguard membrane systems from the detergent-like effects of free acyl-CoAs (42). Free acyl-CoAs, consisting of a carboxylic acid group attached to a coenzyme A (CoA), exert detergent-like properties that can cause structural disturbance to lipid membranes through regulation of their release to enzymes that utilize them. In summary, it is known that CHAT has a strong relationship with AD and even neurological disorders, and previous studies have shown that early AD can be diagnosed by measuring CHAT levels in peripheral blood. ACBD6 does not seem to have much association with AD but is highly associated with human lipid homeostasis and lipid metabolism. However, it should not be overlooked that ACBD6 can be associated with many neurological symptoms through its involvement in cellular metabolism. It proposed that both CHAT and ACBD6 are related to AD in one way or another.

Inflammation is a key factor in AD progression, and it may worsen the pathological changes in AD by producing cytokines that affect the production and clearance of Aβ and tau (43). Some immune factors interact with Aβ peptide and may influence AD progressions, such as risk genes, complement, microglia, monocytes, and lymphocytes (44). The AD group has a high expression of helper T-cells and monocytes, while naive B-cell expression is lower than in the normal group. Notably, monocytes are crucial for Aβ clearance. Their activation state and cytokine secretion determine whether they have a beneficial or detrimental effect.

Nonetheless, the interaction between Cephaelin Hydrochloride, Ainsliadimer A, Gypsogenin3-O-beta-D-glucuronide 6’-methyl ester, Aristolochic acid A, and lipid metabolism remains unknown. Non-coding RNA (miR-342-3p, miR-146a, miR-195, miR-9, and miR-15) is involved in the development of Alzheimer’s disease. Notably, these RNA are linked to A accumulation (45). It is unknown if the expected gene-targeted medications and non-coding RNA can have a role, and the particular pathways require more investigation. As a result, the selected medications and non-coding RNA should be explored in the future. CHAT might be a potential target for improving peripheral blood flow or the brain immunological microenvironment in Alzheimer’s disease patients.

CircRNA KIAA1586 has been identified as a critical risk factor in Alzheimer’s Disease (AD), acting as a competing endogenous RNA (ceRNA) through its binding to AD-associated microRNAs (miRNAs) (46). Dysregulated circRNA-mediated ceRNA networks in AD mouse models are implicated in critical biological processes, such as axonal terminal formation, synaptic regulation, amyloid-beta (Aβ) clearance, and myelin function (47). Comprehensive analysis of circRNA-associated ceRNA networks in AD models highlights their significant roles in neuron projection development, cellular morphogenesis, and cranial development (48). Elucidating these ceRNA interactions offers deeper insights into the underlying mechanisms of AD and facilitates the development of innovative diagnostic and therapeutic approaches.

Finally, the marker gene for gene-targeted Chinese medicine and the ceRNA network were assessed. Vitexin has shown potential in regulating neuroinflammation, which is a key factor in AD progression (49). It may have pharmacoepigenetic properties that help in reducing neuroinflammation. Sesamin has demonstrated neuroprotective effects by reducing Aβ toxicity and preventing Aβ oligomerization in a Caenorhabditis elegans model (50). Sesamin also exhibits antioxidative, anti-inflammatory, and antiapoptotic actions, which contribute to its neuroprotective effects in AD models (51). Oroxylin A, along with baicalein and wogonin, has been found to reduce Aβ-induced oxidative stress and inflammation by modulating the NF-κB/MAPK pathway, thereby protecting neurons from apoptosis (52). These findings suggest that these compounds could be potential candidates for developing therapeutic agents for AD. The application for the rest of the six drugs for AD or neurological diseases had not been reported. In terms of the relationship with lipid metabolism, Oroxylin, Ecliptasaponin A, Succinic Acid, and Sesamin can regulate the turnover of lipid droplets in hepatocytes by regulating the expression of enzymes involved in lipid metabolism, such as adipose triglyceride lipase (ATGL) and fatty acid synthase (FAS) (53–57). Vitexin can also mediate lipid metabolism and attenuate lipid accumulation in the liver by activating autophagy and reducing endoplasmic reticulum stress (58).

The interaction between Cephaelin Hydrochloride, Aristolochic Acid A, Gypsogenin 3-O-beta-D-glucuronide 6’-methyl ester, and lipid metabolism hasn’t been thoroughly understood, though. MiR-342-3p, miR-146a, miR-195, miR-9, and miR-15 are non-coding RNAs that are crucial in the development of AD. Notably, these RNA have a strong connection to the buildup of AD [62]. It is uncertain if the projected gene-targeted medications and non-coding RNA can be involved, and more research into the precise mechanisms is required. Therefore, prospective studies on the chosen medicines and non-coding RNA are necessary.

### Study strengths and limitations

Insights into prospective treatment strategies for AD were gained from this study’s identification of potential Chinese medicine medications that particularly target the discovered marker genes. The main flaw in this work is that experimental validation of the suggested biomarkers was not included; instead, it was restricted to the examination of publically accessible gene expression data. While in silico approaches, including machine learning and bioinformatics analyses, provide powerful tools for identifying potential biomarkers and therapeutic compounds, they are inherently limited by the quality and representativeness of the input data. The findings are based on existing datasets and predictive models, which may not fully capture the complex biological interactions in Alzheimer’s disease (AD). Additionally, the study’s sample size was somewhat limited, which would restrict how far the results can be applied. However, comparable results were found using other techniques and cohorts, which increases the reliability and stability of the findings. However, further research is required to emphasize the need for further validation of the identified diagnostic biomarkers (CHAT, RAB4A, ACBD6, GLA) and TCM compounds through *in vitro* and *in vivo* studies. *In vitro* studies should focus on verifying the expression and functional relevance of these genes in AD-related cellular models, particularly in neuronal or glial cell lines.

## Conclusion

This research has identified several genes potentially involved in lipid metabolism in Alzheimer’s disease, including CHAT, RAB4A, ACBD6, and GLA. Among these genes, CHAT is of particular interest as it plays a role in lipid metabolism and may be involved in regulating the brain immune microenvironment in AD patients. Finding these prospective biomarkers is an important step toward a better understanding of the pathophysiology and treatment of Alzheimer’s disease, even if it should be highlighted that gene expression does not always exactly equate to protein expression. These biomarkers can potentially be used in clinical settings to improve early detection and diagnosis of AD, allowing for timely intervention and treatment. While the identification of lipid metabolism-related genes (LMGs) and their association with Alzheimer’s disease (AD) provides valuable insights, it is crucial to emphasize the need for experimental validation to confirm these findings.

## Data Availability

The original contributions presented in the study are included in the article/**Supplementary Material**. Further inquiries can be directed to the corresponding authors.
